# The Protection of the Successful

**Published:** 1912-01-13

**Authors:** 


### The Hospital

The Workers' Newspaper of Administrative Medicine and Institutional Life, Administration, National Insurance and Health.

No. 1331, Vol. LI. SATURDAY, JANUARY 13th, 1912. PRICE ONE PENNY.

### THE PROTECTION OF THE SUCCESSFUL.

One effect of the National Insurance Act has been that most members of the medical profession have been put on their mettle and are justly alarmed at the moment. It is said that its effect upon parents who have had sons anxious to enter the medical schools this year has been disastrous, for many men who were down to enter for the medical curriculum have withheld their names. These sons and their parents are now considering what other profession they shall enter, where the prospects of making an adequate income are free from the dangers which threaten the general body of practitioners throughout the country. To mention this is to prove that, whatever may be said—and certainly much may be properly urged in favour of a carefully thought out system of National Insurance—no statesman or man of business can defend or support the hurry and absence of knowledge which have characterised the forcing of the present Act through Parliament. Enthusiasm is a potent force, but it can only be utilised for honest good where it rests upon the solid basis of thoughtful, adequate, knowledgeable care that the means employed are wise and prudent, while they safeguard to the fullest possible extent the interests of everybody who may be affected, directly or indirectly, by the legislation which results from it. No one doubts the enthusiasm of Mr. Lloyd George, but even fewer doubt, whatever their politics, that he is the last man who ought to have been placed in charge of the finances of a great nation. However much a man may sympathise with his objects, no man of business and no statesman can justify his methods, or the callous disregard which they exhibit for facts, principles, and knowledge, in the absence of which sound finance and wise legislation are impossible.

The profession of medicine has been approaching the parting of the ways with giant strides during the last decade. The tendency in the direction of the destruction of the science of diagnosis, by the free use of the knife, is much to be regretted, and, unless the leaders of the profession take counsel and call a halt, one branch of the medical profession, and some of the most important and essential rights of the patient, may be sacrificed. There was a time when the practice was that no operation should be undertaken by a surgeon except in the presence of a physician, and with the latter's consent. From the communications which constantly reach us, and from the general trend of professional and public opinion, we have come to the conclusion, if the art of medicine is to continue as an honoured profession, and not to degenerate into a mere trade, that it it essential the leaders shall put their heads together and formulate a modern code of ethics, which will be binding upon all members of the medical profession.

The growth of surgical interference and the enormous improvement in technical method and facility and in the skill exhibited by the operator, with the consequent excellence of many of the results to the patients who have placed themselves in the hands of a modern surgeon, have rendered it possible for some members of the profession to earn incomes far beyond those which were formerly within the reach of the most eminent practitioners. It has so come to pass that a successful surgeon may find himself in the position of a man who has so many patients clamouring for his individual services, irrespective of the large fees which he may be able to command, that success has embraced him with a rapidity which has proved dangerous rather than advantageous. Success, after all, in any branch of life, if the interests of the individual are to be promoted by it, must rest upon principles of prudence, patience, and perseverance. The newspapers have published an instance where a fortune of £40,000 gained by hard literary work was speedily lost. The profession can supply instances where enormous success and the sudden command of apparently unlimited supplies have urged the individual forward more and more, until the constant devotion to work has superseded even the ability with which that work was done, and has ended a career of splendid attainment and hard-earned reputation with a suddenness, so tragic in all its consequences, as to be terrible indeed.

The truth is that every man who finds himself moving onwards to success should associate with himself someone of strong character as a friend in whom he can place absolute confidence. He should then entrust to that friend the responsibility of applying the brake and keeping a calm and prudent eye upon all his proceedings and affairs, so that the surgeon may be able to devote his whole attention to the work, and be prevented, if necessary, from overworking and under-resting. Nowadays the motor enables a consultant in a few hours to see five or six patients resident in a wide area, where formerly it was possible to see only one. The fees are the same or even larger than formerly in many cases, so that the temptation to try and get more and more work done in a given time is greater than ever before. The fact is, the greater the facility of accomplishment in the field of work, the stronger is the necessity for caution in so organising it, that the pressure which results to the practitioner may be minimised and he may be safeguarded from the dangers by which his strength and health must otherwise be sapped. Facilities for work, organisation and methods of work, the economy of one's time, the employment of every possible facility to render accomplishment as easy as possible, commendable and necessary as they are, require the recognition of the limitations of the strength and capacity of even the strongest man, however robust and however able. We have taken the present occasion, when such thoughts may profitably be indulged in, to write thus plainly with the assured conviction that the circumstances of the day demand counsels of prudence, especially where individual success may prove to be so rapid and all-engrossing as to become dangerous to individual practitioners.

